# Associations of physical fitness with cortical inhibition and excitation in adolescents and young adults

**DOI:** 10.3389/fnins.2024.1297009

**Published:** 2024-04-29

**Authors:** Hanna Mari Skog, Sara Määttä, Laura Säisänen, Timo A. Lakka, Eero A. Haapala

**Affiliations:** ^1^Department of Physiology, Institute of Biomedicine, School of Medicine, University of Eastern Finland, Kuopio, Finland; ^2^Department of Clinical Neurophysiology, Diagnostic Imaging Center, Kuopio University Hospital, Kuopio, Finland; ^3^Department of Technical Physics, University of Eastern Finland, Kuopio, Finland; ^4^Kuopio Research Institute of Exercise Medicine, Kuopio, Finland; ^5^Department of Clinical Physiology and Nuclear Medicine, Kuopio University Hospital, University of Eastern Finland, Kuopio, Finland; ^6^Faculty of Sport and Health Sciences, University of Jyväskylä, Jyväskylä, Finland

**Keywords:** transcranial magnetic stimulation, motor threshold, motor fitness, muscular strength, adolescence

## Abstract

**Objective:**

We investigated the longitudinal associations of cumulative motor fitness, muscular strength, and cardiorespiratory fitness (CRF) from childhood to adolescence with cortical excitability and inhibition in adolescence. The other objective was to determine cross-sectional associations of motor fitness and muscular strength with brain function in adolescence.

**Methods:**

In 45 healthy adolescents (25 girls and 20 boys) aged 16–19 years, we assessed cortical excitability and inhibition by navigated transcranial magnetic stimulation (nTMS), and motor fitness by 50-m shuttle run test and Box and block test, and muscular strength by standing long jump test. These measures of physical fitness and CRF by maximal exercise were assessed also at the ages 7–9, 9–11, and 15–17 years. Cumulative measures of physical measures were computed by summing up sample-specific *z*-scores at ages 7–9, 9–11, and 15–17 years.

**Results:**

Higher cumulative motor fitness performance from childhood to adolescence was associated with lower right hemisphere resting motor threshold (rMT), lower silent period threshold (SPt), and lower motor evoked potential (MEP) amplitude in boys. Better childhood-to-adolescence cumulative CRF was also associated with longer silent period (SP) duration in boys and higher MEP amplitude in girls. Cross-sectionally in adolescence, better motor fitness and better muscular strength were associated with lower left and right rMT among boys and better motor fitness was associated with higher MEP amplitude and better muscular strength with lower SPt among girls.

**Conclusion:**

Physical fitness from childhood to adolescence modifies cortical excitability and inhibition in adolescence. Motor fitness and muscular strength were associated with motor cortical excitability and inhibition. The associations were selective for specific TMS indices and findings were sex-dependent.

## Introduction

Physical fitness encompasses motor fitness, muscular strength, and cardiorespiratory fitness (CRF), and refers to an individual’s ability to perform physical activity (Caspersen et al., 1985). Physical fitness is considered an important marker of metabolic and vascular health (Ortega et al., 2008a), and it has also been positively related to brain health, especially cognition, in children and adolescents (Donnelly et al., 2016). Furthermore, previous studies utilizing structural and functional magnetic resonance imaging (MRI) have found positive associations of overall physical fitness or CRF with hippocampal volume (Chaddock et al., 2010), entorhinal cortex gray matter volume (Whiteman et al., 2016), and whole-brain functional connectivity (Talukdar et al., 2018) in children and young adults. Electrophysiological brain imaging studies have also observed that higher CRF is associated with better top-down control and cortical communication (Hsieh et al., 2020) and inhibitory control (Mora-Gonzalez et al., 2020) in children and young adults. However, the evidence on the associations of physical fitness with cortical inhibitory and excitatory balance in adolescents remains limited. A better understanding of the role of physical fitness in motor cortical inhibitory and excitatory balance is important because adolescence is a sensitive and one of the most important life periods for brain development after early childhood (Herting and Sowell, 2017).

Neuronal activity is coordinated through the balance of inhibitory and excitatory inputs. The primary function of excitatory neurons is to integrate and transmit information within and between brain areas (Wood et al., 2017). Inhibitory interneurons, in turn, shape the way excitatory neurons integrate information (Wood et al., 2017) and are involved, for example, in the modulation of neural plasticity (Daskalakis et al., 2007). The associations of physical fitness with cortical inhibitory and excitatory inputs can be studied by transcranial magnetic stimulation (TMS), a non-invasive method to examine excitation-inhibition balance within cortical circuits (Nicolini et al., 2021). TMS offers direct neurophysiological insight into the excitatory and inhibitory networks and neurotransmitter functioning as well as functional connectivity within neural circuits (Ziemann et al., 2015; Nicolini et al., 2021) and allows a unique way to examine cortical excitation, which cannot be obtained with any other neuroimaging methodologies (Hervé et al., 2009). TMS is also well-suited for examining developmental and lifestyle-related changes in brain functioning (Kaarre et al., 2016; Määttä et al., 2017).

Previous TMS studies have mainly focused on the associations of aerobic exercise with cortical excitability and inhibition in adults (Cirillo et al., 2009; McDonnell et al., 2013). A single bout of physical exercise has been found to modify cortical excitability and promote motor cortex plasticity (Moscatelli et al., 2021). However, the associations of physical fitness with brain functions are scarce, particularly from longitudinal data. Therefore, the associations of physical fitness with cortical excitability and inhibition remain uncertain (Nicolini et al., 2019, 2021). Although the evidence suggests positive cross-sectional associations of physical fitness with brain structures and functions in children, there are no previous studies on the associations of physical fitness with cortical excitatory and inhibitory regulatory systems in adolescents.

We investigated the longitudinal associations of cumulative motor fitness, muscular strength, and CRF from childhood to adolescence with cortical excitability and inhibition in adolescence adjusted by age. We also studied the cross-sectional associations of motor fitness and muscular strength with cortical excitability and inhibition in adolescence. Due to sex differences in the rate of maturation, physical fitness, and brain functions (Blackmore et al., 2010; Armstrong et al., 2011), we examined these associations separately in girls and boys. Finally, we investigated the modifying effect of body fat percentage (BF%) on the associations of physical fitness with cortical excitability and inhibition, because adiposity is strongly associated with indices of physical fitness (Haapala et al., 2016), and it has been associated with impaired cognitive development in children and adolescents (Herting and Sowell, 2017).

## Materials and methods

### Study design and participants

The current analyses are based on data from the Physical Activity and Nutrition in Children (PANIC) study (Lakka et al., 2020; Sallinen et al., 2022) and its neuroimaging sub-study FitBrain. The PANIC study design, participants, and protocol have been described in detail previously (Lakka et al., 2020; Sallinen et al., 2022). Briefly, the PANIC study is an 8-year lifestyle intervention study in a general population of 504 children aged 7–9 years at the baseline examinations in 2007–2009. Of these children, 439 participated in the 2-year follow-up examinations in 2009–2011 at the age of 9–11 years, and 277 attended the 8-year follow-up examinations in 2015–2017 at the age of 15–17 years. These 277 adolescents constituted the study group, from which the participant for the FitBrain study was invited. The adolescents who did not participate in the 8-year follow-up assessments did not differ in sex or pubertal stage distribution, age, height, weight, body mass index standard deviation score, performance in the Box and block test (BBT), or CRF [W_max_/lean body mass (LM)] at baseline or 2-year follow-up from those who participated in the 8-year follow-up (all *p* > 0.100 for the difference). Those who did not participate in the 8-year follow-up assessments had lower BF% at baseline (mean difference = −2.1, 95% CI = −3.6 to −0.7) and 2-year follow-up (mean difference = −2.7, 95% CI = −4.6 to −1.0), poorer standing long jump performance at 2-year follow-up (mean difference = 5.7, 95% CI = 1.6 to 9.8), and worse shuttle run test performance at 2-year follow-up (mean difference = −0.5, 95% CI = −0.8 to −0.1), and were more often from families with lower parental education levels at baseline (*p* < 0.001) and 2-year follow-up (*p* = 0.027) than those who participated in the 8-year follow-up assessments.

**FIGURE 1:**
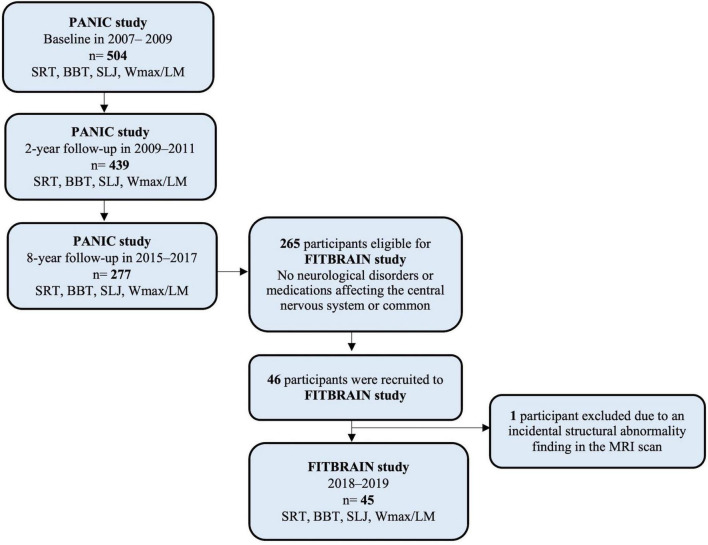
Flow chart of the PANIC study. SRT, shuttle run test; BBT, Box and block test; SLJ, standing long jump; W_max_/LM, maximal work load scaled by lean body mass.

Of the adolescents participating in the 8-year follow-up examinations of the PANIC study, 265 had complete data on motor fitness, muscular strength, body size, and body composition (see details on these assessments below) and had no neurological disorders or medications affecting the central nervous system or common contraindications to TMS (Rossi et al., 2021). The FitBrain study participants were recruited from adolescents who participated in the 8-year follow-up examinations of the PANIC study, and altogether 46 adolescents aged 16–19 years participated in the FitBrain study in 2018–2019 (Figure 1). The adolescents who participated in the FitBrain study did not differ in sex or pubertal stage distribution, age, height, weight, body mass index standard deviation score, BF%, physical fitness, or parental education from other adolescents who participated in the 8-year follow-up assessment of the PANIC study. One adolescent with an incidental finding of structural brain abnormality in the MRI scan was excluded. Therefore, the final sample used in the current analyses included 45 youth (25 girls and 20 boys). Adolescents who were included in the analyses were moderately physically active as they accumulated 111.0 (±35.9), 117.2 (±34.6), and 137.0 (±73.9), minutes of questionnaire-assessed physical activity (PA) per day at baseline, 2-year follow-up, and 8-year follow-up, respectively. In the PANIC study, motor fitness, muscular strength, CRF, body size, and body composition were assessed at baseline (2007–2009), at 2-year follow-up (2009–2011), and 8-year follow-up (2015–2017) (Table 1). In the FitBrain study, cortical inhibition and excitation balance were assessed in 2018–2019. We also assessed motor fitness, muscular strength, body size, and body composition in the FitBrain study.

**TABLE 1:** Measures in different PANIC study stages.

Measures	PANIC study stages			
	0 year	2 years	8 years	FitBrain
**Body size:** body weight (kg), body height (cm)	x	x	x	x
**Body composition:** body fat percentage (%), lean body mass (kg), body mass index (kg/m^2^)	x	x	x	x
**Motor fitness:** the 50-m shuttle run test (time), the Box and block test (number of cubes)	x	x	x	x
**Muscular strength:** standing long jump test (cm)	x	x	x	x
**Cardiorespiratory fitness:** maximal workload (W_max_) scaled by lean body mass	x	x	x	
**Brain functions**				
**Resting motor threshold** (% of maximum stimulator output), **cortical silent period** (ms), **silent period threshold** (% of maximum stimulator output), **long-interval intracortical inhibition** (%), **motor evoked potential amplitude** (μV)				x

The Research Ethics Committee of the Hospital District of Northern Savo approved the PANIC study (Statements 69/2006 and 295/2015) and the FitBrain study (Statement 366/2017). Prior to the PANIC study, the parents or guardians of the children gave their written informed consent, and the children provided their assent to participation. The adolescents also gave their written informed consent prior to the 8-year follow-up examinations of the PANIC study and later prior to the FitBrain study. The PANIC study and the FitBrain study have been carried out in accordance with the principles of the Declaration of Helsinki.

### Assessment of body size and composition

Body height (cm) was measured using a calibrated wall-mounted stadiometer using standard procedures. Body weight (kg) was measured using the Inbody 720^®^ bioimpedance device (Biospace, Seoul, Republic of Korea) using the standard protocol (Eloranta et al., 2012). Body fat percentage (BF%) and lean body mass (LM) (kg) were measured using the Lunar^®^ dual-energy X-ray absorptiometry (DXA) device (Lunar Prodigy Advance; GE Medical Systems, Madison, WI, USA), the children being in the non-fasting state, having emptied the bladder, and being in light clothing with all metal objects removed (Tompuri et al., 2015). In the FitBrain study, BF% and LM were measured using the Inbody 720^®^ bioimpedance device (Biospace, Seoul, Republic of Korea), the participant having emptied the bladder and wearing light underwear.

### Assessment motor fitness and muscular strength

Motor fitness and muscular strength were assessed at all four stages of the current study (Table 1). Motor fitness was assessed by the 50-m shuttle run test (SRT) (Adam et al., 1988). The participant was asked to run 5 m from a start line to another line as fast as possible, turn on the line, run back to the start and continue until five shuttles were completed. The test score was the running time in seconds, with a longer time indicating poorer performance.

Manual dexterity as well as upper limb movement skills and speed were assessed by the BBT (Mathiowetz et al., 1985). The test was performed using a wooden box (53.7 cm × 25.4 cm × 8 cm) divided into two identical compartments by a partition. At the beginning of the test, 150 small wooden cubes (2.5 cm/side) were placed in one compartment. The participant was asked to pick up the cubes one by one with the dominant hand from one side of the wooden box and to move as many cubes as possible over the partition to the other side of the box for 60 s. The same task was repeated with the non-dominant hand. The test score was the total number of cubes moved to the other side of the box in 2 min, a smaller number of cubes moved indicating poorer manual dexterity. Handedness was assessed using the Waterloo Handedness Questionnaire (Steenhuis et al., 1990).

Lower limb explosive muscular strength was assessed by the standing long jump (SLJ) test (Adam et al., 1988). The participant was asked to stand with their feet together, jump as far as possible, and land on both feet. The test score was the longest jump of three attempts in centimeters.

### Assessment of cardiorespiratory fitness

Cardiorespiratory fitness was assessed at the baseline, 2-year follow-up, and 8-year follow-up of the PANIC study (Table 1). CRF was assessed by a maximal cycle ergometer test using a pediatric saddle module (Ergoselect 200 K, Ergoline, Bitz, Germany) and was defined as the maximal power output (W_max_) at the end of the exercise test scaled by LM (Tompuri et al., 2015). After 3-min warming up at 5 W, children cycled at a cadence of 70–80 revolutions per min for 1 min at 20 W, and after that, the workload steadily increased at a rate of 1 W per 6 s until voluntary exhaustion. The exercise test was considered maximal if the reason for terminating the test indicated maximal effort and maximal cardiorespiratory capacity. Peak workload was defined as the workload at the end of the test.

### Assessment of brain functions

Brain functions were assessed in the FitBrain study (Table 1). For neuronavigation, the participant was scanned with a 3T scanner (Philips Achieva TX, Philips Healthcare, Eindhoven, the Netherlands) for structural three-dimensional T1-weighted MR-images (TR 8.07 ms, TE 3.7 ms, flip angle 8°, 1 mm × 1 mm × 1 mm resolution). A neuroradiologist screened the MRI for any abnormalities before the navigated transcranial magnetic stimulation (nTMS) examination. Before nTMS, it was also confirmed that the participants had not performed vigorous physical activity.

Navigated transcranial magnetic stimulation was performed with an eXimia stimulator, (Nexstim Plc., Helsinki, Finland) and a figure-of-eight coil combined with a navigation system version 3.2.2 to enable continuous visualization of the stimulation coil in relation to the individual cortical anatomical structures and ensuring optimal tilting, i.e., placing the coil tangentially to the head. nTMS-induced motor evoked potentials (MEPs) were recorded using disposable Ag-AgCl surface electrodes placed on the abductor pollicis brevis muscle bilaterally using a belly-tendon montage. Throughout the measurement, muscle activity was monitored online and recorded by stimulus-locked electromyography (Nexstim Plc., Helsinki, Finland). First, the optimal cortical representation site (“hotspot”) of the abductor pollicis brevis muscle was determined on both hemispheres (Säisänen et al., 2018). The hotspot was the stimulation site where the MEPs of the highest amplitude were elicited repeatedly.

The following measurements were targeted at the hotspots of abductor pollicis brevis muscle separately on the left and right hemispheres using biphasic nTMS. The individual resting motor threshold (rMT) was determined using a threshold hunting paradigm Motor Threshold Assessment Tool (MTAT) 2.0 (Awiszus and Borckardt, 2012) as a percentage of the maximum stimulator output (%-MSO) with an amplitude limit of 50 μV until the confidence interval was 95%. The rest of the measurements were performed on the left hemisphere only.

Following measurements of inhibition were performed:

• Cortical silent period (SP) duration was measured using a stimulus intensity of 120% of rMT (SP120) with moderate muscle contraction. The participants were provided with online electromyography and asked to use muscle contraction at about half of the maximum force (about 500 μV in amplitude). The task was to squeeze the soft balls simultaneously with both hands. Ten trials were collected. SP120 was assessed as absolute duration: the mean for each participant was calculated after excluding trials with the shortest and longest duration (Säisänen et al., 2008).
• SP threshold (SPt) was determined as the stimulus intensity that produced SP of 30 ms duration, the so-called SPt30 (Kallioniemi et al., 2014). When the duration of the SP exceeded 30 ms, it was assessed as a response, whereas shorter or missing SP duration was determined as no response. MTAT was used to determine the threshold that was considered confident enough after 20 stimuli.

Finally, since paired pulses cannot be performed using biphasic waveform due to technical restrictions of the Nexstim stimulator, the following measurements were performed at the previously determined hotspot using monophasic TMS. rMT for monophasic TMS was measured similarly as described above for biphasic TMS. Thereafter, 80 single pulse MEPs were elicited at 120% rMT. A peak-to-peak amplitude for each single pulse MEP was assessed and the mean value for each subject was calculated (hereafter called “MEP amplitude”). These were used as unconditioned test MEPs for the long-interval intracortical inhibition (LICI) paradigm. Trials with muscle activity were discarded. LICI was assessed using 80 pairs of conditioning and test stimuli set at 120% rMT, an inter-stimulus interval of 100 ms and the inter-trial interval was randomized between 3 and 5 s. LICI was assessed as the mean of each ratio of conditioned to the unconditioned mean test MEP amplitude.

### Other assessments

The parents were asked to report their highest completed or ongoing educational degrees, categorized as vocational school or less, polytechnic, and university, and the degree of the more educated parent was used in the analyses.

### Statistical analyses

All statistical analyses were performed using the IBM SPSS statistics, version 25.0, for Macintosh (IBM Corporation, Armonk, NY, USA). The normality of variable distributions was tested with the Kolmogorov–Smirnov test and visually from histograms. Differences and associations with p-values <0.05 were considered statistically significant. Differences in characteristics between boys and girls were compared using the independent samples t-test for normally distributed variables, the Mann–Whitney U-test test for variables with skewed distributions, or with Chi-square test for categorical variables. Because of the skewed distributions, we performed logarithmic transformation for MEP amplitude and LICI median before the linear regression analyses.

Cumulative physical fitness scores and cumulative BF% across baseline, 2-year follow-up, and 8-year follow-up of the PANIC study, representing the longitudinal exposure to physical fitness and BF%, were computed separately for each individual measure of physical fitness and BF% and using the sample specific z-scores and the formula:

$$Z-s\,c\,o\,r\,e_{b\,a\,s\,e\,l\,i\,n\,e}+Z\text{-}s\,c\,o\,r\,e_{2\text{-}y\,e\,a\,r\,f\,o\,l\,l\,o\,w\text{-}u\,p}+Z\text{-}s\,c\,o\,r\,e_{8\text{-}y\,e\,a\,r\,f\,o\,l\,l\,o\,w\text{-}u\,p}$$

For example, cumulative 50-m SRT performance was computed by summing up the time spent to complete the test (transformed to sample specific *z*-scores) at baseline, 2-year follow-up, and 8-year follow-up, as:

$$Z_{t\,i\,m\,e^{b\,a\,s\,e\,l\,i\,n\,e}}+Z_{t\,i\,m\,e^{2-y\,e\,a\,r\,f\,o\,l\,l\,o\,w-u\,p}}+Z_{t\,i\,m\,e^{8-y\,e\,a\,r\,f\,o\,l\,l\,o\,w-u\,p}}$$

We analyzed the longitudinal associations of these cumulative scores for 50-m SRT, BBT, and SLJ test performances, peak workload (W_max_) from the baseline, 2-year follow-up, and 8-year follow-up of the PANIC study with the TMS measures (including rMTs, SP120, SPt, LICI, and MEP amplitude) from the FitBrain study using linear regression analyses separately for boys and girls adjusted for age. Age was entered into the linear regression model in Step 1, and the measures of physical fitness were entered separately into the models in Step 2. These longitudinal data were additionally adjusted for the cumulative score for BF% to analyze whether adiposity confounds those associations. Unadjusted associations between the measures of physical fitness and TMS variables are presented visually in the Supplementary Figure 1.

We analyzed the cross-sectional associations of 50-m SRT, BBT, and SLJ test performances with TMS variables, all from the FitBrain study, using linear regression analyses separately for boys and girls adjusted for age. These cross-sectional data were additionally adjusted for BF% to analyze whether adiposity confounded the associations.

All linear regression models were checked for linearity using the scatter plots and normal distribution and homoscedasticity of residuals using the normal probability plots and residual scatter plots, respectively (see Supplementary material for example). Additionally, we examined the possibility of multicollinearity in our models using the variance inflation factor (VIF) and tolerance. We used a threshold value of 5 for VIF and 0.2 for tolerance.

The data were corrected for multiple comparisons using the Benjamini–Hochberg false discovery rate (FDR) using the FDR value of 0.25. We considered standardized regression coefficients between 0.10 and 0.29, between 0.30 and 0.49 are medium, and ≥0.50 to describe small, medium, and large effect sizes, respectively.

## Results

### Basic characteristics and nTMS measure of participants in the FitBrain study

Boys were taller and heavier and had higher LM, lower BF%, and larger head circumference than girls (Table 2). Boys also were faster in the 50-m SRT and jumped a longer distance in the SLJ test but were slower in the BBT at all four stages of the PANIC study compared to girls (Supplementary Table 1). All participants were right-handed (Table 2). There were no statistically significant differences in any nTMS measure between boys and girls (Table 3).

**TABLE 2:** Characteristics of participants in the FitBrain study.

	All (*N* = 45)	Girls (*N* = 25)	Boys (*N* = 20)	*p*-value
Age (year)	17.9 ± 0.8	17.8 ± 0.8	18.0 ± 0.8	0.354
Height (CM)	172.1 ± 9.1	165.8 ± 4.6	179.9 ± 6.6	<**0**.**001**
Weight (KG)	67.1 ± 11.2	60.8 ± 8.4	74.9 ± 9.0	<**0**.**001**
Body mass index (kg/m^2^)	22.6 ± 3.0	22.01 ± 3.0	23.2 ± 2.8	0.202
Lean body mass (kg)	50.0 ± 10.4	42.4 ± 5.7	59.5 ± 6.4	<**0**.**001**
Body fat percentage (%)	21.2 ± 7.7	25.6 ± 5.4	15.7 ± 6.4	<**0**.**001**
Head circumference (CM)	56.8 ± 5.8 (56.5)	55.9 ± 1.1 (56.0)	58.1 ± 1.6 (58.0)	<**0**.**001**
Handedness (scored from −40 to +40)*	26.2 ± 5.8 (26)	27.2 ± 5.7 (26)	24.9 ± 5.8 (26)	0.198
Parental education (%)				0.672
Vocational school or less	14.3	12.5	16.7	
Polytechnic	33.3	29.2	38.9	
University	52.4	58.3	44.4	

The data are means and their standard deviations for normally distributed variables and medians and interquartile ranges for variables with skewed distributions. Differences in characteristics between boys and girls were analyzed using the independent samples *t*-test for normally distributed variables, the Mann–Whitney U-test for variables with skewed distributions, and Chi-square test for categorical variables. Statistically significant (*p* < 0.05) differences between girls and boys are indicated by bolded *p*-values. *Scored from fully left-handed (−40) to fully right-handed (40), ambidextrous subjects scoring from −15 to 15.

**TABLE 3:** Transcranial magnetic stimulation variables in all participants and in girls and boys separately.

	All (*N* = 45)	Girls (*N* = 25)	Boys (*N* = 20)	*p*-value
rMT left (%)	42.0 ± 7.3 [30–59]	40.4 ± 7.9 [30–59]	43.9 ± 6.1 [35–59]	0.111
rMT right (%)	42.3 ± 7.1 [27–60]	40.7 ± 7.6 [27–59]	44.3 ± 5.7 [37–60]	0.094
rMT mono left (%)	63.3 ± 10.5 [46–94]	61.9 ± 9.9 [46–83]	65.0 ± 11.2 [49–94]	0.343
SP120 duration (ms)	67 ± 26 [24–115]	67 ± 28 [24–115]	67 ± 23 [24–111]	0.866
SPt (%)	39.0 ± 6.8 [28–54]	37.0 ± 7.0 [28–54]	41.0 ± 6.0 [29–53]	0.061
MEP amplitude (μV)*	833 ± 1006 [238–4369]	1031 ± 888 [238–3273]	784 ± 1158 [374–4369]	0.564
LICI median*	0.00 [0.00–0.34]	0.00 [0.00–0.34]	0.00 [0.00–0.15]	0.669

The data are means and their standard deviations (ranges) for normally distributed variables (rMT left, rMT right, rMT, mono SP120, and SPt), medians and their ranges for variables with skewed distributions (MEP amplitude and LICI median). Differences in variables between girls and boys were analyzed using the independent samples *t*-test for normally distributed variables and using the Mann–Whitney U-test for variables with skewed distributions. rMT, resting motor threshold; LICI, long-interval cortical inhibition; SPt, cortical silent period threshold; MEP, motor evoked potential. *For MEP amplitude and LICI median, there were 41 adolescents (23 girls and 18 boys).

### Longitudinal associations of average motor fitness, muscular strength, and CRF from childhood to adolescence with brain functions in adolescence

All linear regression models met the assumptions for linearity as well as the normal distribution and homoscedasticity of residuals. Furthermore, VIF was <5 and tolerance >0.2 in all models.

In boys, a better cumulative 50-m SRT performance from childhood to adolescence was associated with lower rMT bilaterally and a lower MEP amplitude in adolescence after adjustment for age at baseline (Table 4). Furthermore, a better childhood-to-adolescence cumulative BBT performance was associated with lower rMT bilaterally in adolescence (Table 4), but its association with rMT at the left hemisphere was not statistically significant after further adjustment for childhood-to-adolescence cumulative BF% (Supplementary Table 2). A better cumulative 50-m SRT performance from childhood to adolescence was associated with lower SPt and higher MEP amplitude in adolescence adjusted for age at baseline (Table 4), but these associations were no longer statistically significant after additional adjustment for childhood-to-adolescence average BF% (Supplementary Table 2). Higher cumulative CRF from childhood to adolescence was associated with longer SP120 in adolescence (Table 4), and this association remained statistically significant after further adjustment for childhood-to-adolescence cumulative BF% (Supplementary Table 2).

**TABLE 4:** Longitudinal associations of cumulative motor fitness, muscular strength, and cardiorespiratory fitness from childhood to adolescence with brain functions in adolescence adjusted for age.

	rMT at left hemisphere		rMT at right hemisphere		LICI		SP120		SPt		MEP amplitude	
50-m	β	*p*	β	*p*	β	*p*	β	*p*	β	*p*	β	*p*
**SRT**												
Girls	-0.222	0.331	-0.198	0.381	-0.061	0.800	0.344	0.121	-0.105	0.653	-0.157	0.512
Boys	**0**.**862**	<**0**.**001**	**0**.**844**	<**0**.**001**	-0.024	0.937	0.002	0.995	**0**.**670**	**0**.**009**	**0**.**646**	**0**.**017**
**BBT**												
Girls	-0.030	0.906	-0.083	0.743	-0.134	0.615	-0.309	0.211	0.066	0.794	0.333	0.188
Boys	-**0**.**585**	**0**.**010**	-**0**.**788**	<**0**.**001**	-0.046	0.857	-0.006	0.788	-0.430	0.070	-0.018	0.946
**SLJ**												
Girls	-0.193	0.391	-0.035	0.874	-0.082	0.728	0.361	0.097	-0.323	0.150	0.003	0.990
Boys	-0.123	0.669	-0.281	0.323	0.481	0.075	-0.290	0.297	-0.077	0.789	-0.255	0.387
**W_max_/LM**												
Girls	-0.089	0.761	-0.263	0.268	-0.300	0.229	0.099	0.681	-0.054	0.822	**0**.**602**	**0**.**008**
Boys	-0.177	0.505	-0.299	0.293	-0.353	0.173	**0**.**532**	**0**.**029**	0.394	0.125	0.269	0.331

The data are standardized regression coefficients and their *p*-values from linear regression analyses adjusted for age at baseline. Statistically significant (*p* < 0.05) associations are indicated by bolded standardized regression coefficients and *p*-values. SRT, shuttle run test; BBT, Box and block test; SLJ, standing long jump; W_max_/LM, maximal workload scaled by lean body mass (LM); rMT, resting motor threshold; LICI, long-interval cortical inhibition; SP120, cortical silent period duration using a stimulus intensity of 120% of resting motor threshold; SPt, cortical silent period threshold; MEP, motor evoked potential.

In girls, higher cumulative CRF from childhood to adolescence was associated with higher MEP amplitude in adolescence (Table 4), and this association remained statistically significant after further adjustment for childhood-to-adolescence cumulative BF% (Supplementary Table 2).

Cumulative 50-m SRT, BBT, or SLJ performance from childhood to adolescence was not associated with LICI in adolescence after adjustment for age at the baseline and childhood-to-adolescence cumulative BF% in either sex (Supplementary Table 2). Moreover, childhood to adolescence cumulative CRF was not associated with rMT at the left or right hemisphere, LICI or SPt in adolescence after adjustment for age and cumulative BF% in girls or boys.

### Cross-sectional associations of motor fitness and muscular strength with brain functions in adolescents

In boys, a better performance in the 50-m SRT and the SLJ test were associated with lower rMT bilaterally (Table 5). A shorter 50-m SRT time and longer SLJ distance were also associated with a lower rMT at the right hemisphere. However, further adjustment for BF% attenuated the associations of the 50-m SRT and SLJ performance with rMT at the left hemisphere, but their associations with rMT at right hemisphere remained materially unchanged (Supplementary Table 3). The positive association between 50-m SRT performance and SPt was not statistically significant after adjustment for age (Table 5), but further adjustment for BF% increased the magnitude of this association making it statistically significant (Supplementary Table 3). Similarly, the association between better BBT performance and lower rMT at the right hemispheres, and the association with a shorter 50-m SRT time and a longer SP120 became statistically significant after further adjustment for BF% (Supplementary Table 3).

**TABLE 5:** Cross-sectional associations of motor fitness and muscular strength with brain functions in adolescence adjusted for age in adolescence.

FitBrain	rMT at left hemisphere		rMT at right hemisphere		LICI		SP120		SPt		MEP amplitude	
50-m	β	*p*	β	*p*	β	*p*	β	*p*	β	*p*	β	*p*
**SRT time**												
Girls	0.251	0.339	0.178	0.431	0.132	0.573	-0.067	0.770	0.275	0.230	-0.033	0.887
Boys	**0**.**509**	**0**.**020**	**0**.**651**	<**0**.**001**	0.025	0.923	-0.153	0.511	0.420	0.053	-0.198	0.448
**BBT**												
Girls	0.040	0.856	-0.012	0.956	-0.094	0.685	-0.132	0.549	0.084	0.706	**0**.**437**	**0**.**044**
Boys	-0.436	0.074	-0.459	0.058	-0.099	0.726	-0.136	0.587	-0.466	0.058	-0.148	0.589
**SLJ**												
Girls	-**0**.**444**	**0**.**040**	-0.314	0.141	-0.192	0.410	0.127	0.579	-**0**.**578**	**0**.**006**	0.001	0.997
Boys	-**0**.**478**	**0**.**031**	-**0**.**747**	<**0**.**001**	0.265	0.302	-0.136	0.560	-0.157	0.491	0.106	0.685

The data are standardized regression coefficients and their *p*-values from linear regression analyses adjusted for age in adolescence. Associations with *p*-values < 0.05 are bolded. SRT, shuttle run test; BBT, Box and block test; SLJ, standing long jump; rMT, resting motor threshold; LICI, long-interval cortical inhibition; SP120, cortical silent period duration; SPt, cortical silent period threshold; MEP, motor evoked potential.

In girls, a better BBT performance was associated with a larger MEP amplitude (Table 5). This association remained statistically significant after further adjustment for BF%. Also a longer SLJ distance was associated with a lower rMT at the right hemispheres, although this association was no longer statistically significant after further adjustment for BF% (Supplementary Table 3). A better result in the SLJ test was associated with a lower SPt and also this association remained statistically significant after further adjustment for BF% (Supplementary Table 3). None of the motor fitness or muscular strength test results was associated with LICI after adjustment for age and BF%, neither in girls nor in boys (Supplementary Table 3).

## Discussion

We found that higher levels of cumulative motor fitness and muscular strength from baseline to 8-year follow-up were associated with greater motor cortical excitability and stronger motor cortical inhibition in adolescence. However, these associations were only observed for specific TMS indices. Additionally, we observed that these associations were sex-dependent. These findings were also generally confirmed in the cross-sectional analyses. Furthermore, cumulative CRF across the follow-up was associated with some indices of motor cortical excitability and inhibition in adolescence.

Longitudinally, better cumulative motor fitness performance from childhood to adolescence was associated with lower right hemisphere rMT, lower SPt, and lower MEP amplitude in boys. Better childhood-to-adolescence cumulative CRF was associated with longer SP duration in boys and higher MEP amplitude in girls. Cross-sectionally in adolescence, better motor fitness and better muscular strength performance were associated with lower left and right rMT among boys and better motor fitness performance was associated with higher MEP amplitude and better muscular strength performance with lower SPt among girls. In general, our results aligns with previous findings (Mora-Gonzalez et al., 2020; Moscatelli et al., 2021), showing that children and adolescents with better physical fitness differ in brain functions from their peers with poorer physical fitness.

While the associations of physical fitness with cortical excitability and inhibition were partly different in boys and girls, the reasons for these differences are unclear. Explanations may include differences in maturation and the rate of neurodevelopment (Säisänen et al., 2018) as well as the volume and intensity of habitual physical activity (El-Sayes et al., 2020; Nicolini et al., 2021) between boys and girls.

### Associations of motor fitness, muscular strength, and cardiorespiratory fitness with cortical excitability

In the present study, cortical excitability was assessed by rMT and MEP amplitude. MT represents cortico-cortical axon excitability and suprathreshold MEP amplitude reflects transsynaptic activation of corticospinal neurons and the initial axon segments of corticospinal neurons (Ziemann et al., 2015). The excitatory circuits network is probably regulated by ionotropic glutamatergic, GABAergic, and neuromodulating neurotransmitters (Ziemann et al., 2015; Hupfeld et al., 2020).

We observed that better motor fitness and muscular strength were cross-sectionally associated with a lower rMT among adolescent boys and better motor fitness performance with higher MEP amplitude in girls. Some previous exercise-based TMS studies have investigated the effects of acute bouts of exercise modulate corticospinal excitability or neuroplasticity, but mostly among healthy, physically active individuals and the findings of these studies are partly mixed (Nicolini et al., 2021). Monda et al. (2017) found that 25 karate athletes (25 ± 4.9 years) with high motor fitness had increased cortical excitability, as indicated by a lower rMT and a higher MEP amplitude than 25 age-matched non-athletes. Moscatelli et al. (2020) found that longer aerobic training intervention decreased rMT in 15 (24 ± 2.1 years) healthy sedentary participants. Similarly, an increased MEP amplitude has been found in response to motor training in 14 active young adults (24 ± 4 years) compared to 14 sedentary participants (Cirillo et al., 2011). In line with these findings, our results indicate that exercise training which enhances motor fitness also might improve brain functions in adolescence.

We found that better cumulative CRF from childhood to adolescence was associated with a higher MEP amplitude among girls in adolescence, but not among adolescent boys. In previous studies (Moscatelli et al., 2020), 8 weeks of aerobic exercise training improved CRF, decreased rMT, and increased MEP amplitude among 15 healthy young men (24 ± 2.1 years), while Nicolini et al. (2019) found no changes in corticospinal excitability after 6 weeks of aerobic interval training in 18 sedentary men (23 ± 3.5 years), although the training improved CRF. Our results together with previous findings (e.g., Nicolini et al., 2019), suggest that longer exposure to exercise may be needed to induce beneficial changes in cortical excitability.

### Associations of motor fitness, muscular strength, and CRF with cortical inhibition

We assessed cortical inhibition by SP duration, SP threshold, and LICI. The early part of SP duration reflects spinal inhibition and the late part of SP supraspinal, likely motor cortical postsynaptic inhibition (Ziemann et al., 2015; Hupfeld et al., 2020). Longer durations are mediated by the action of the gamma-amino butyric acid receptor, type B (GABA_BR_)-action. In contrast shorter durations are mediated by type A (GABA_AR_)-action (Ziemann et al., 2015). SP threshold indicates the stimulus intensity needed for the induction of SP (Kallioniemi et al., 2014) and LICI describes postsynaptic GABA_BR_ activity (Valls-Sole et al., 1992).

In our cross-sectional analyses, we found that girls with better muscular strength had lower SPt than weaker girls. Moreover, boys with better cumulative motor fitness from childhood to adolescence had lower SPt in adolescence, than boys with poorer cumulative motor fitness. We also observed that a higher cumulative CRF from childhood to adolescence was associated with longer SP duration among boys in adolescence. In previous studies in adults, CRF has not been associated with GABA concentration in the motor cortex (Nicolini et al., 2019; Harasym et al., 2020). To the best of our knowledge, we observed for the first time an inverse association between CRF and motor cortical excitability and a direct association between CRF and motor cortical inhibition among adolescents. Few studies in adults have examined the effects of long-term exercise training on corticospinal excitability and intracortical circuit functioning using TMS (Nicolini et al., 2021). Mooney and coworkers observed that an acute aerobic exercise bout decreased LICI but did not affect SP duration in adults (Mooney et al., 2016). However, we found neither cross-sectional nor longitudinal associations of motor fitness, muscular strength, or CRF with LICI.

Silent period duration seems to increase with growing fatigue during aerobic exercise, but the effect is not similar to long-term participation in exercise (Nicolini et al., 2021). One explanation for the inconsistent observations may be that the modulation of cortical inhibition by physical activity mediated by GABA_BR_ and GABA_AR_, depends on the duration and intensity of exercise (Nicolini et al., 2021). Although acute aerobic exercise has not been found to alter SP duration, it is possible that our observation that higher cumulative CRF from childhood to adolescence was associated with a longer SP duration among boys in adolescence reflects adaptation to physical activity from childhood, and suggests that boys with better fitness had stronger GABA_BR_ cortical inhibition (Ziemann et al., 2015). Physical fitness affects mechanisms of excitability and inhibition that are important for normal brain development and maturation (Talukdar et al., 2018). While the role of cortical inhibition in brain functions is not entirely understood, recent neuropsychological studies have observed associations of GABA_BR_ markers with mental health in adolescents (Lewis et al., 2018).

### Associations modified by adiposity

Body fat percentage modified part of the associations of motor fitness, muscular strength, and CRF with brain functions. The findings of previous studies suggest that increased adiposity and related cardiometabolic risk factors could negatively influence the brain (Lamballais et al., 2018; Ortega et al., 2019; Mora-Gonzalez et al., 2020). Therefore, it would not be surprising that the measures of motor fitness and muscular strength, which are strongly related to body fat mass, could have similar pathways influencing the brain than adiposity. Nevertheless, we found that some associations of motor fitness, muscular strength, and CRF with brain functions were independent of BF% in boys. Moreover, adjustment for BF% strengthened some associations of motor fitness with brain functions, suggesting that BF% could adjust these associations. Therefore, our results and previous findings, suggest that physical fitness may be important for brain development independent of adiposity even though adiposity could modify the associate. The data in the FitBrain study was not adjusted for socioeconomic factors since the majority of the participants came from families with high parental education levels. It is worth noting that the PANIC study, from which the FitBrain participants were recruited, also included families with higher parental education than the original PANIC study sample.

### Strengths and limitations

The strengths of our study include valid and reproducible measures of motor fitness, muscular strength, and CRF from childhood to adolescence and the assessment of cortical excitation and inhibition using nTMS in adolescence. Our data allowed us to investigate cross-sectional associations of physical fitness with cortical excitation and inhibition in adolescence and to study whether average physical fitness from childhood to adolescence modifies cortical excitation and inhibition. However, we did not measure CRF at the same time point as brain functions, but we used the CRF data collected earlier in the PANIC study. We also used W_max_ as a measure of CRF instead of directly measuring maximal oxygen uptake. While using directly measured maximal oxygen uptake would have been optimal, W_max_ is an acceptable surrogate measure of maximal oxygen uptake in youth (Dencker et al., 2008; Haapala et al., 2024). In addition, although the research staff made every effort to encourage the participants to give their best in the fitness tests, which are considered reliable (Platz et al., 2005; Ortega et al., 2008b; Fjørtoft et al., 2011; Pate et al., 2012), we cannot completely rule out that the motivation, given effort, and day-to-day variation in the physical fitness tests did not influence the observed association. Furthermore, we assessed cortical excitation and inhibition only once in adolescence (FitBrain study). Therefore, we could not investigate the parallel development of physical fitness and brain functions. At the final time point (FitBrain study), we used the bioelectrical impedance method instead of DXA to assess BF% and LM. Therefore, the results of the longitudinal and cross-sectional analyses may not be directly comparable. However, DXA and bioelectrical impedance methods have been found to have acceptable agreement (Sillanpää et al., 2014; Tompuri et al., 2015). Moreover, the sample size was relatively small, especially in sex-stratified analyses and larger studies with better statistical power are needed to confirm our findings and to test whether our findings can be generalized to larger populations of youth. The data in the FitBrain study was not adjusted for socioeconomic factors since most of the participants came from families with high parental education levels. It is also worth noting that the study population at the 8-year time point of the PANIC study, from which the FitBrain participants were recruited, also included families with higher parental education than the original PANIC study sample. Because socioeconomic status has been associated with physical fitness and brain development, it is possible that high socioeconomic status in our study sample decreased the magnitude of the associations between physical fitness and brain functions. Accordingly, our results may not be generalizable to populations with lower socioeconomic status. Finally, we could not provide evidence for the causality of the associations of motor fitness, muscular strength, and CRF with cortical excitation and inhibition.

## Conclusion

We found that better physical fitness was associated with lower motor cortical excitability and stronger motor cortical inhibition in adolescents. However, these associations were sex-dependent and selective for TMS indices. Our results, in general, suggest that improving physical fitness influences cortical inhibition and excitation balance, and physical fitness modulates the regulation of networks and functions in the brain among adolescents. However, intervention studies on the effects of exercise training on physical fitness and cortical excitability and inhibition in adolescents and young adults are warranted.

## Data availability statement

The data that support the findings of this study are not openly available due to reasons of sensitivity and are available from the corresponding author upon reasonable request.

## Ethics statement

The studies involving humans were approved by the Research Ethics Committee of the Hospital District of Northern Savo. The studies were conducted in accordance with the local legislation and institutional requirements. Written informed consent for participation in this study was provided by the participants’ legal guardians/next of kin.

## Author contributions

HS: Formal analysis, Funding acquisition, Investigation, Methodology, Project administration, Visualization, Writing – original draft. SM: Data curation, Funding acquisition, Investigation, Project administration, Resources, Supervision, Writing – review & editing. LS: Data curation, Funding acquisition, Investigation, Supervision, Writing – review & editing. TL: Data curation, Funding acquisition, Investigation, Project administration, Resources, Supervision, Writing – review & editing. EH: Data curation, Formal analysis, Funding acquisition, Investigation, Project administration, Resources, Supervision, Writing – review & editing.
