# Supplementary material for: Associations of physical fitness with cortical inhibition and excitation in adolescents and young adults
Source: Front Neurosci. 2024 Apr 29;18:1297009. doi: 10.3389/fnins.2024.1297009 (PMC11090042; doi:10.3389/fnins.2024.1297009)
Supplement: Supplementary file 1 [file Table_1.docx]

| **Table S1. Motor fitness, muscular strength, and cardiorespiratory fitness in all participants and in girls and boys separately** | | | | | | | | | | | | |
| --- | --- | --- | --- | --- | --- | --- | --- | --- | --- | --- | --- | --- |
|  | **PANIC study stages** | | | | | | | | | | | |
|  | **Baseline in 2007- 2009** | | | **2-year follow-up in 2009-2011** | | | **8-year follow-up in 2015-2017** | | | **FITBRAIN study in 2018-2019** | | |
|  | **All**  n=42–44 | **Girls**  n=23 | **Boys**  n=18–20 | **All**  n=40–44 | **Girls**  n=23–24 | **Boys**  n=17–20 | **All**  n=42–45 | **Girls**  n=22–25 | **Boys**  n=19–20 | **All**  n=45 | **Girls**  n=25 | **Boys**  n=20 |
| **50-meter SRT performance (s)** | 24.4 (2.2) | 24.5 (2.3) | 24.2 (2.4) | 21.7 (1.6) | 21.9 (1.6) | 21.3 (1.7) | 20.6 (1.9) | 21.5 (1.7) | 19.5 (1.4) | 20.5 (1.9) | 21.4 (1.7) | 19.3 (1.5) |
| **BBT performance (cubes moved)** | 102 (13.2) | 105 (12.5) | 97 (13.2) | 118 (12.2) | 122 (11.7) | 113 (11.2) | 137 (16.7) | 137 (15.5) | 136 (18.4) | 151 (15.5) | 154 (14.8) | 147 (15.9) |
| **SLJ test**  **performance (cm)** | 124 (14.8) | 119 (13.4) | 129 (14.9) | 146 (23.8) | 139 (17.6) | 156 (27.7) | 193 (33.3) | 172 (23.8) | 220 (22.8) | 197 (36.9) | 174 (24.7) | 226 (28.1) |
| **W_max_/LM** | 3.58 (0.5) | 3.36 (0.5) | 3.84 (0.5) | 4.37 (0.5) | 4.26 (0.4) | 4.51 (0.4) | 4.51 (0.5) | 4.69 (0.4) | 4.27 (0.6) |  |  |  |
| The data are means and their standard deviations.  CRF= Cardiorespiratory fitness, SRT = Shuttle run test, BBT = Box and block test, SLJ = Standing long jump, W_max_/LM = maximal work load scaled by lean body mass | | | | | | | | | | | | |
