# Supplementary material for: Associations of physical fitness with cortical inhibition and excitation in adolescents and young adults
Source: Front Neurosci. 2024 Apr 29;18:1297009. doi: 10.3389/fnins.2024.1297009 (PMC11090042; doi:10.3389/fnins.2024.1297009)
Supplement: Supplementary file 2 [file Table_2.docx]

| Table S2. Longitudinal associations of average motor fitness, muscular strength, and cardiorespiratory fitness from childhood to adolescence with brain functions in adolescence after adjustment for age and cumulative adiposity from childhood to adolescence. | | | | | | | | | | | | | | |
| --- | --- | --- | --- | --- | --- | --- | --- | --- | --- | --- | --- | --- | --- | --- |
|  | **rMT at left hemisphere** | | **rMT at right**  **hemisphere** | | **LICI** | | **SP120** | | **SPt** | | | **MEP amplitude** | | |
| 50-meter SRT  Girls  Boys | β  -0.204  **0.945** | p  0.420  **0.027** | β  -0.102  **0.896** | p  0.682  **0.037** | β  -0.048  0.595 | p  0.854  0.288 | β  -0.186  -0.233 | p  0.464  0.646 | | β  -0.193  0.711 | p  0.434  0.155 | | β  -0.345  0.444 | p  0.484  0.237 |
| BBT  Girls  Boys | 0.006  -0.423 | 0.980  0.145 | -0.060  **-0.753** | 0.812  **0.003** | -0.094  -0.484 | 0.722  0.154 | 0.201  0.230 | 0.447  0.583 | | 0.094  -0.292 | 0.702  0.343 | | 0.436  -0.037 | 0.150  0.567 |
| SLJ  Girls  Boys | -0.142  0.419 | 0.629  0.277 | 0.047  0.152 | 0.872  0.700 | -0.030  0.440 | 0.918  0.278 | 0.354  -0.264 | 0.230  0.501 | | -0.163  0.441 | 0.558  0.228 | | 0.056  0.139 | 0.773  0.257 |
| W_max_/LM  Girls  Boys | -0.169  -0.034 | 0.480  0.903 | -0.334  -0.197 | 0.155  0.457 | -0.229  -0.416 | 0.369  0.153 | 0.153  **0.570** | 0.536  **0.046** | | -0.180  -0.262 | 0.422  0.336 | | **0.688**  0.340 | **0.003**  0.323 |
| The data are standardized regression coefficients and their p-values from linear regression analyses adjusted for age in childhood and cumulative body fat percentage (%). Associations with *p* -values <0.05 are bolded. SRT = shuttle run test, BBT = Box and Block test, SLJ = standing long jump, W_max_/LM = maximal work load scaled by lean body mass (LM), rMT = resting motor threshold, LICI = long-interval cortical inhibition, SP120 = Cortical silent period duration, SPt = cortical silent period threshold, MEP = motor evoked potential. | | | | | | | | | | | | | | |
