# Supplementary material for: Associations of physical fitness with cortical inhibition and excitation in adolescents and young adults
Source: Front Neurosci. 2024 Apr 29;18:1297009. doi: 10.3389/fnins.2024.1297009 (PMC11090042; doi:10.3389/fnins.2024.1297009)
Supplement: Supplementary file 3 [file Table_3.docx]

| Table S3. Associations of motor fitness and muscular strength with brain functions in adolescence after adjustment for age and adiposity in adolescence | | | | | | | | | | | | | |
| --- | --- | --- | --- | --- | --- | --- | --- | --- | --- | --- | --- | --- | --- |
| FitBrain | **rMT left**  **hemisphere** | | **rMT right hemisphere** | | **LICI** | | **SP120** | | **SPt** | | **MEP amplitude** | |  |
| 50-meter SRT time  Girls  Boys | β  -0.050  0.532 | p  0.818  0.087 | β  -0.040  0.376 | p  0.859  0.127 | β  -0.073  0.271 | p  0.768  0.439 | β  -0.005  **-0.779** | p  0.985  **0.007** | β  0.003  **0.816** | p  0.989  **0.007** | β  -0.120  -0.129 | p  0.939  0.844 |  |
| BBT  Girls  Boys | 0.026  -0.392 | 0.888  0.100 | -0.023  **-0.369** | 0.905  **0.044** | -0.104  -0.126 | 0.632  0.665 | -0.129  -0.090 | 0.564  0.715 | 0.070  -0.450 | 0.707  0.066 | **0.561**  0.085 | **0.047**  0.535 |  |
| SLJ  Girls  Boys | -0.250  -0.509 | 0.238  0.135 | -0.144  **-0.591** | 0.510  **0.020** | -0.017  0.331 | 0.945  0.417 | 0.082  0.267 | 0.749  0.442 | **-0.390**  -0.328 | **0.037**  0.365 | -0.023  -0.077 | 0.826  0.700 |  |
| The data are standardized regression coefficients and their p-values from linear regression analyses adjusted for age and body fat percentage (%) InBody in adolescence. Associations with p-values <0.05 are bolded. SRT = shuttle run test, BBT = Box and Block test, SLJ = Standing long jump, rMT = resting motor threshold, LICI = long interval cortical inhibition, SP120 = cortical silent period duration, SPt = cortical silent period threshold, MEP = motor evoked potential. | | | | | | | | | | | | |  |
