# Supplementary material for: Associations of physical fitness with cortical inhibition and excitation in adolescents and young adults
Source: Front Neurosci. 2024 Apr 29;18:1297009. doi: 10.3389/fnins.2024.1297009 (PMC11090042; doi:10.3389/fnins.2024.1297009)
Supplement: Supplementary file 4 [file Table_4.DOCX]

**Supplementary Figure 2. A**n example of a normal probability plot (A) and residual scatter plots (B) to investigate the normal distribution and homoscedasticity of residuals. These example plots are from the linear regression analyses in boys on the associations between cumulative Box and block test and resting motor threshold at the right hemisphere adjusted for age*.* The linear regression slope of the homoscedasticity plot (B) did not differ from zero (p>0.9999).
