# Supplementary figures and images for: Associations of physical fitness with cortical inhibition and excitation in adolescents and young adults
Source: Front Neurosci. 2024 Apr 29;18:1297009. doi: 10.3389/fnins.2024.1297009 (PMC11090042; doi:10.3389/fnins.2024.1297009)

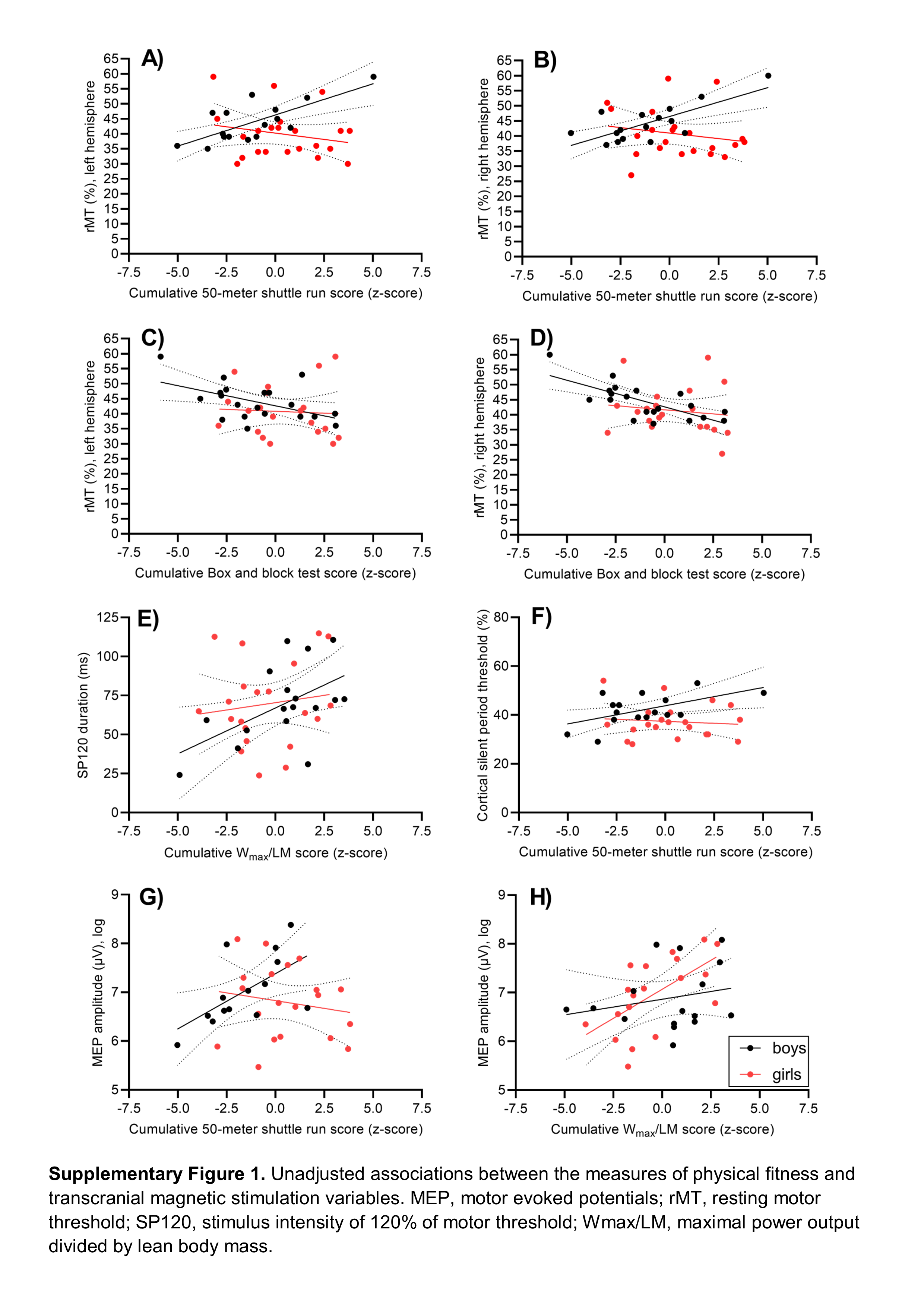

Supplement: Supplementary file 5 [file Image_1.tif]

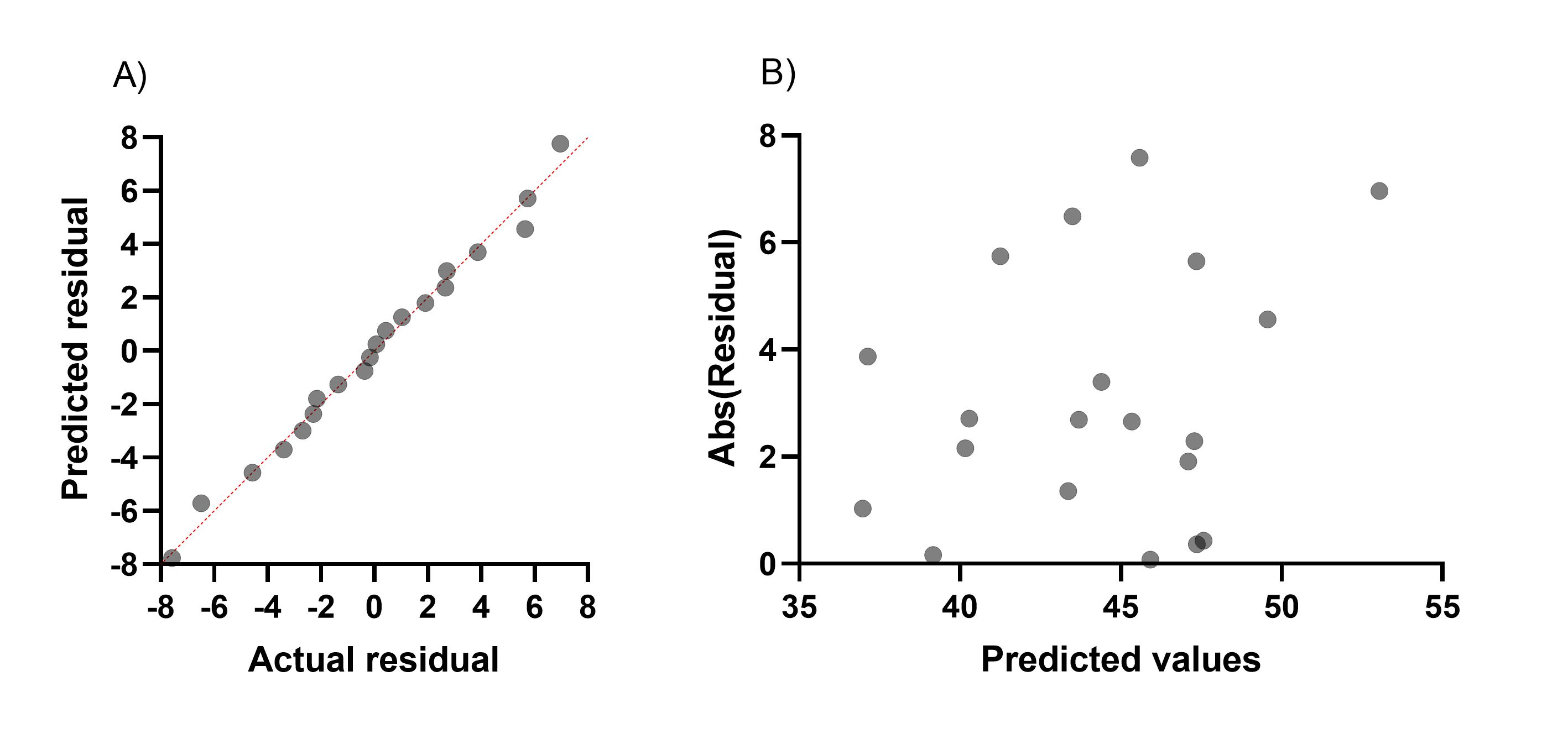

Supplement: Supplementary file 6 [file Image_2.TIFF]
